# Dyrk1A Is Dynamically Expressed on Subsets of Motor Neurons and in the Neuromuscular Junction: Possible Role in Down Syndrome

**DOI:** 10.1371/journal.pone.0054285

**Published:** 2013-01-16

**Authors:** Gloria Arque, Anna Casanovas, Mara Dierssen

**Affiliations:** 1 Genes and Disease Program, Center for Genomic Regulation (CRG), Pompeu Fabra University, Barcelona Biomedical Research Park (PRBB) and CIBER de Enfermedades Raras (CIBERER), Barcelona, Spain; 2 Department of Experimental Medicine and IRBLLEIDA, University of Lleida, School of Medicine, Lleida, Catalonia, Spain; IGBMC/ICS, France

## Abstract

Individuals with Down syndrome (DS) present important motor deficits that derive from altered motor development of infants and young children. *DYRK1A*, a candidate gene for DS abnormalities has been implicated in motor function due to its expression in motor nuclei in the adult brain, and its overexpression in DS mouse models leads to hyperactivity and altered motor learning. However, its precise role in the adult motor system, or its possible involvement in postnatal locomotor development has not yet been clarified. During the postnatal period we observed time-specific expression of Dyrk1A in discrete subsets of brainstem nuclei and spinal cord motor neurons. Interestingly, we describe for the first time the presence of Dyrk1A in the presynaptic terminal of the neuromuscular junctions and its axonal transport from the facial nucleus, suggesting a function for *Dyrk1A* in these structures. Relevant to DS, *Dyrk1A* overexpression in transgenic mice (TgDyrk1A) produces motor developmental alterations possibly contributing to DS motor phenotypes and modifies the numbers of motor cholinergic neurons, suggesting that the kinase may have a role in the development of the brainstem and spinal cord motor system.

## Introduction

Down syndrome (DS) is the most frequent aneuploidy leading to mental retardation [1]. Motor dysfunction is highly prevalent among individuals with DS, who exhibit clumsy sequences of movements [2], and poor control in programming motor sequences, their timing and force [3]. Delays in achieving motor development milestones are also a constant feature in DS children [4], [5]. Babies and young children with DS are late to reach motor milestones such as grasping, rolling, sitting, standing and walking. Some of those DS-associated motor deficits have been reproduced in some mouse models [6], [7] and could be contributed by malfunctioning of the cerebellum [6], [8], [9].

Some candidate genes have been related to DS phenotypes, but few of those could be related to motor dysfunction. *DYRK1A* (dual-specificity tyrosine- (Y)-phosphorylation-regulated kinase 1A) is located on HSA21 [10]. It encodes for a serine threonin kinase, that belongs to an evolutionarily conserved family of proteins involved in functions generally related with growth and development [11].

The role of *DYRK1A* in pathways controlling motor function is supported by its expression pattern and the motor phenotype observed in heterozygous and transgenic *Dyrk1A* mice (TgDyrk1A). Dyrk1A is expressed ubiquitously in the developing nervous system but in adult brain its expression is confined to neurons of the olfactory bulb, the cerebellar cortex, the spinal cord and motor nuclei of the brainstem [12], [13], [14]. However, little is known of the concrete role of Dyrk1A in motor function or its expression in the postnatal period. This is relevant to DS-related motor dysfunction, since DS motor phenotypes derive to some extent from delays in postnatal motor development. TgDyrk1A overexpressing Dyrk1A show significant levels of motor dysfunction in tasks involving coordination, motor learning and organization of motor behavior [15], [16], [17] that resemble to some extent the motor phenotypes present in DS patients. Also, both TgDyrk1A and a partial trisomy mouse model (Ts65Dn) of DS present a delay in neuromotor development [15], although this has not been reproduced in a BAC transgenic mouse strain overexpressing human *DYRK1A* [18]. In addition, reduced *Dyrk1A* expression in heterozygous *Dyrk1A* mice leads to a clear hypoactivity [19], [20]. Even though altered motor function in DS has been mainly ascribed to dysfunction in the cerebellum, other brain structures, such as the motor nuclei in the brainstem or the spinal cord or the striatum are also crucial in the generation and control of motor behavior. Interestingly, intrastriatal injections of viral vectors expressing shRNA against *Dyrk1A* into TgDyrk1A mice rescued their motor defects [17] suggesting that Dyrk1A expression in various brain regions may contribute to the DS motor alterations.

Here we report time-specific expression of Dyrk1A in discrete subsets of brainstem nuclei and spinal cord motor neurons during the period of craniocaudal motor maturation. Dyrk1A is transported to the presynaptic neuromuscular junction as revealed by experimental axotomy, suggesting a role for the kinase in neuromuscular junction formation and/or function. Relevant to DS, *Dyrk1A* overexpression in transgenic mice (TgDyrk1A) produces motor developmental alterations possibly contributing to DS motor phenotypes. Also Dyrk1A overexpression modifies the numbers of motor cholinergic neurons, suggesting that the kinase may have a role in the development of the brainstem and spinal cord motor system.

## Materials and Methods

### Animals and General Procedures

In order to study the contribution of *DYRK1A* to Down syndrome phenotypes, we used a single gene transgenic mouse model overexpressing *Dyrk1A* (TgDyrk1A) [15]. The non-transgenic littermates of TgDyrk1A mice served as controls. TgDyrk1A is a single-gene transgenic mouse overexpressing specifically the full-length cDNA of *Dyrk1A* under the control of sheep metallothionein-Ia (*sMT-Ia*) promoter in a C57BL6/SJL genetic background. The genotyping was performed by PCR analysis using the primer pair: DYRK-forward primer, 5′-GTC CAA ACT CAT CAA TGT ATC-3′ and DYRK-reverse primer, 5′-CTT GAG CAC AGC ACT GTT G-3′. Each cycle (32 cycles) consisted of 94°C for 30 s, 52°C for 30 s and 72°C for 45 s. The levels of this transcript were higher in the transgenic mice, in the range of ∼1.5–2.2 fold increased. Same-sex littermates were group housed under a 12 h light/dark schedule (lights on at 7∶00 a.m.) in controlled environmental conditions of humidity (60%) and temperature (22±1°C) with free access to food and water.

### Ethics Statement

All animal procedures were approved by the local ethical committee (Comité Ético de Experimentación Animal del PRBB (CEEA-PRBB); procedure numbers MDS-08-1060P1 and JMC-07-1001P1-MDS), and met the guidelines of the local (law 32/2007) and European regulations (EU directive n° 86/609, EU decree 2001-486) and the Standards for Use of Laboratory Animals n° A5388-01 (NIH). The CRG is authorized to work with genetically modified organisms (A/ES/05/I-13 and A/ES/05/14). All efforts were done to minimize animal suffering.

### Behavioral Studies

All the pregnant dams were allowed to deliver spontaneously. The day of delivery was designated as postnatal day 0 (PD0) of age of the neonates. On delivery, the litter size of each dam was recorded and each pup was checked for gross abnormalities. Litters were nursed with their natural dams until weaning at PD21. The pups (wild type: n = 36; TgDyrk1A: n = 39) mice were individually marked with ink at PD7. Behavioral studies to assess neurological and psychomotor development were performed according to Dierssen et al. [21]. Briefly, neuromotor development was assessed on PD7, PD10, PD14 and PD21 by pivoting locomotion test, walking activity, negative geotaxis, climbing ability and wire suspension test. Each experimental day, before the behavioral testing pups were weighed and body/tail length measured, too. Pivoting is an immature locomotion pattern defined by rotations around the hind limbs. Pivoting locomotion test was performed on a flat surface with lines drawn to delineate four 90° segments. The number of pivoting (measured as 90° turns) in 60 seconds was recorded. The ability to walk in a straight line is an indicative of a mature neuromotor development. In the walking test, the latency to walk was measured as the time the mouse waits until it starts moving in a straight line for a distance equal or higher than its own length in less than 60 seconds. To measure negative geotaxis, the mouse is placed on a metallic grid of 45° slope with its head pointing down. It will turn around and crawl up the slope. The score was defined as following: (0) no response, (1) the animal turns around but it doesn’t climb, and (2) the animal turns 180° and climb up. The climbing ability was measured when the pup is held against a vertical metallic grid (wire: 0.6 mm in diameter, mesh: 6 mm wide). One behavior is scored as (0) if there is not response and (1) if the animal shows the ability to climb. In the wire suspension test, pups were placed on a wire (4 mm diameter) in an upside-down position. The ability of the animal to remain suspended was measured as the latency to fall. The maximum time allowed was 60 seconds. All the behavioral testing was conducted by the same experimenter in an isolated room and at the same time of the day. Behavioral experimenters were blinded to the genetic status of the animals.

### Tissue Preparation and Immunohistochemical Procedures

Mice (PD7, PD10, PD14, PD21 and adults; minimum n = 5 per group) were deeply anesthetized with CO_2_ and transcardially perfused with physiological saline solution followed by 4% paraformaldehyde (Sigma, St Louis, MO, USA) in 0.1 M phosphate buffer (PB; pH 7.4). Brain, spinal cord and muscle (caudal portion of digastricus and stylohyoideus muscle) were removed and immersed overnight in fresh fixative at 4°C. Processing of the tissue was different depending on the experiment. Part was transferred to 30% sucrose in 0.1 M PB for 24 hours and frozen, while the rest was embedded in paraffin after dehydration with increasing concentrations of ethanol until arriving to xylene. Frozen spinal cord and brain were sliced in coronal sections (16 µm) whereas muscles were cut into longitudinal sections with the aid of a cryostat.

Tissue sections were processed using streptavidin-biotin-peroxidase complex immunohistochemical method or by immunofluorescence. In both cases, sections were incubated with 10% fetal bovine serum (FBS) and then incubated with the primary antibody overnight at 4°C. Primary antibodies against Dyrk1A (diluted 1∶500, [12] and 1∶200 AbNova Corporation, Tebu, France), serotonin (diluted 1∶35000, DiaSorin, Stillwater, MN, USA), tyrosine hydroxilase (TH, diluted 1∶8000, Sigma, St Louis, MO, USA), choline acetyltransferase (ChAT, diluted 1∶400, Chemicon, Temeluca, CA, USA), Calbindin D-28K (diluted 1∶200, Sigma, St Louis, MO, USA), Calretinin (diluted 1∶500, Sigma, St Louis, MO, USA), Parvalbumin (diluted 1∶500, Sigma, St Louis, MO, USA), vesicular glutamate transporter 1 (vGlut1, diluted 1∶2500, Chemicon, Temeluca, CA, USA), vesicular glutamate transporter 2 (vGlut2, diluted 1∶1000, Synaptic Systems, Göttingen, Germany), glutamate decarboxylase (GAD_65–67_, diluted 1∶500, Chemicon, Temeluca, CA, USA) and calcitonin gene related peptide (CGRP, diluted 1∶1000, Sigma, St Louis, MO, USA) were used. The sections were then incubated with the appropriate biotinylated secondary antibody or with secondary antibodies labeled either with Alexa Fluor 488 or Alexa Fluor 546 (Molecular Probes, Eugene, OR, USA) for multifluorescent labeling. In the first case, the sections were then incubated with the biotinylated link and the streptavidin-HRP as indicated in the manufacturer’s instructions (DAKO, LSAB system, Ely, UK). Peroxidase activity was visualized with 0.05% diaminobenzidine and 0.01% hydrogen peroxide. Sections were dehydrated with increasing concentrations of ethanol until arriving to xylene, and cover slipped with DPX (distyrene, tricresyl phosphate, and xylene; BDH Laboratory Supplies, UK). For immunofluorescence, labeled sections were mounted using Vectashield Mounting medium with DAPI (Vector Laboratories, Burlingame, CA, USA) and stored at 4°C.

Skeletal muscle sections (caudal portion of digastricus and stylohyoideus muscle) that are innervated by facial nerve (VII) were directly incubated with Alexa Fluor 488-conjugated α-bungarotoxin (α-Btx, 1 µg/ml; Molecular Probes, Eugene, OR, USA) to detect the acetylcholine receptors (AchRs) at the neuromuscular synaptic sites and synaptophysin (diluted 1∶100; DakoCytomation Glostrup, Denmark) as a pre-synaptic marker.

The sections were examined using a Leica DMR (Leica Microsystems AG, Wetzlar, Germany) microscope with a Leica DC500 digital camera coupled to the microscope. Immunofluorescence sections were captured on a Leica TCS SPE with appropriate lasers. Intensity analysis was performed on images acquired under identical microscope conditions and software settings. For the final published figures, images were assembled, labeled and balanced for optimal brightness and contrast by using either Adobe Photoshop or ImageJ software (National Institutes of Health, Bethesda, MD, USA).

### Morphology and Stereological Techniques

To construct the morphological maps we used coronal slices (16 µm). Counterstained with the Nissl technique using 0.1% cresyl violet and other markers (Dyrk1A, ChAT) to characterize specific neuronal populations at PD7, PD10 and PD21. Images of the regions of interest were taken, sections were reconstructed and neuronal maps were drawn. Counts of facial motoneurons were performed on serial Nissl stained paraffin sections (9 µm). Adult mice motoneurons of the facial nucleus are localized in a well-defined region of the brainstem that allows counting their cell bodies. Briefly, a minimum of wild type and TgDyrk1A adult (6 months old) mice (n = 6 per genotype), were transcardially perfused as described above. Sections were stained using the Nissl technique with 0.1% cresyl violet, dehydrated with increasing concentrations of ethanol to xylene, and coverslipped with DPX. Large motoneurons, with clearly defined nucleolus, were counted from every fourth section with the aid of a camera lucida. The area of each motoneuron was also measured.

### Axotomy of the Facial Nerve

Adult (6 months old) mice were anesthetized with 3% isoflurane. Using aseptic techniques, the right facial nerve of each animal was exposed and transected at its exit from the stylomastoid foramen [22]. Successful transections were identified by an absence of whisker movement at the left side of the face. The contra lateral side was used as a control. At 2, 7 and 14 days after axotomy, the animals (n = 4 for each time point and genotype) were deeply anesthetized and perfused transcardially as it was described above. The lower brainstem segment was quickly removed and post-fixed in the same fixative. Serial 40 µm thick sections of the brainstem were processed for Dyrk1A immunohistochemistry and for Dyrk1A/CGRP fluorescent labeling, in order to quantify Dyrk1A intensity in the motoneurons of the facial nucleus.

### Data Analysis

Simple comparisons between TgDyrk1A and wild type mice in behavioral tasks and morphological/stereological studies were performed using the two-tailed Student’s t test or repeated measures ANOVA. Quantitative data are expressed as mean ± standard error (S.E.M.). In all tests, a difference was considered significant at p values <0.05. The statistical analysis was performed using the SPSS 17.0 software.

## Results

### Neurobehavioral Development

In order to study the contribution of *DYRK1A t*o Down syndrome phenotypes, we used a transgenic mouse model for *Dyrk1A (T*gDyrk1A) [15]. TgDyrk1A is a single-gene transgenic mouse overexpressing specifically the full-length cDNA of *Dyrk1A* under the control of sheep metallothionein-Ia (*sMT-Ia*) promoter. The levels of this transcript were higher in the transgenic mice, in the range of ∼1.5 to 2.2 fold increased.

No significant differences were detected in weight and body length increase during the preweaning period in wild type (n = 36) versus TgDyrk1A (n = 39) mice (data not shown). TgDyrk1A mice showed no significant differences in the achievements of physical milestones, such as fur appearance, incisor eruption, eyelid opening or permeabilisation of the auditory canal. Also, no differences were detected in sensoriomotor functions, such as forelimb/hind limb placing and grasping, tactile orientation or Preyer’s reflex. These data indicate that *Dyrk1A* overexpression did not affect the overall growth or the acquisition of sensoriomotor functions in mice.

### Neuromotor Development

**Figure 1:**
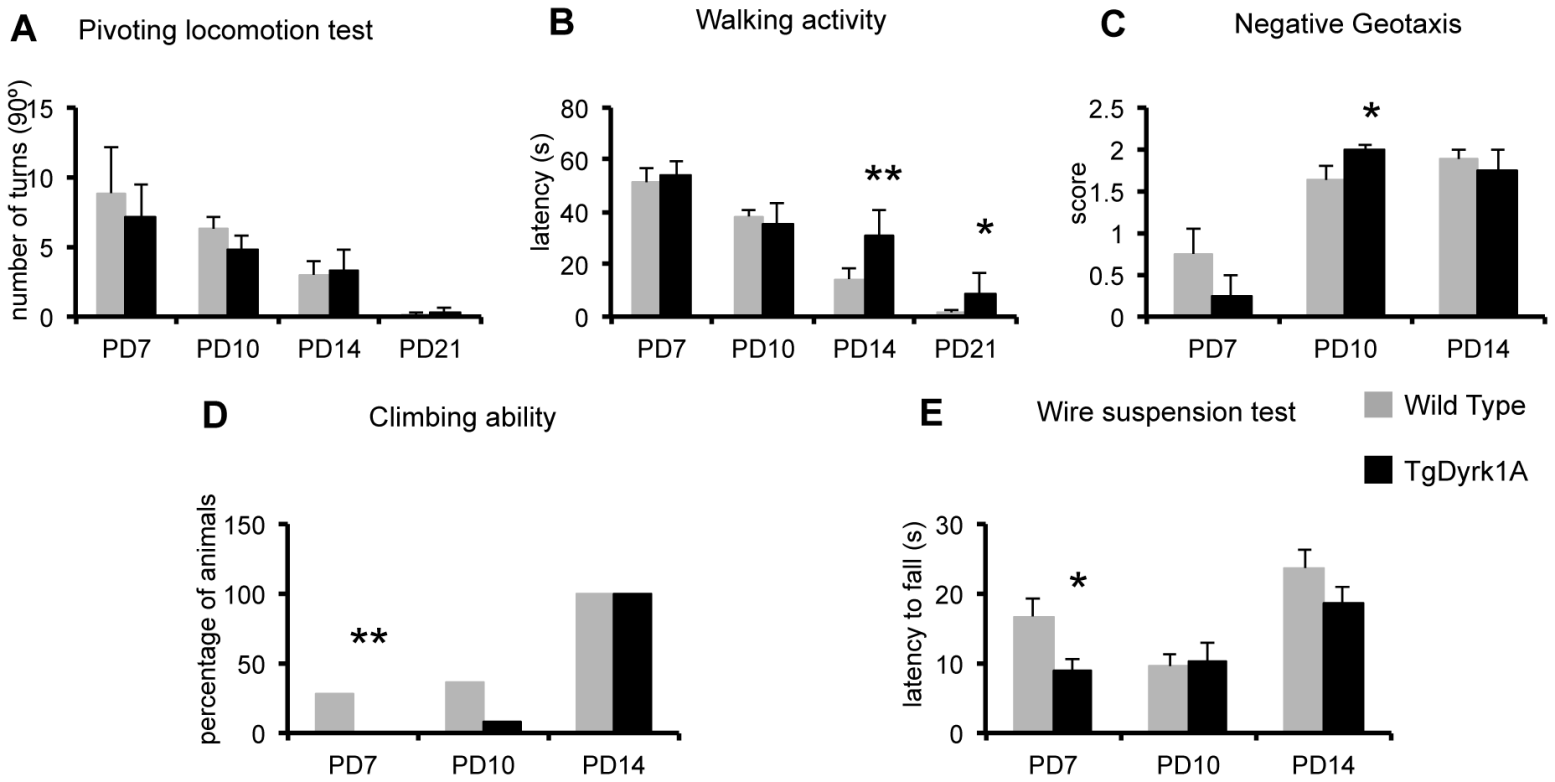
Neuromotor development of TgDyrk1A mice. A) Pivoting activity: TgDyrk1A mice showed a similar number of turns (times the mouse rotated 90 degrees on its hindlimbs) over a 60 sec period in the pivoting test. B) Walking activity: TgDyrk1A mice showed longer latency to initiate walking at PD14 and PD21. C) Negative geotaxis: TgDyrk1A mice exhibited a delay in acquiring negative geotaxis as shown by the impaired performance at PD7 that was recovered at later stages (PD14). D) The percentage of TgDyrk1A mice able to perform the vertical climbing was reduced at PD7 and PD10. E) In the wire suspension test, the latency to fall of TgDyrk1A mice was reduced at P7 developmental stages. Data are presented as mean+SEM, * P<0.05, ** P<0.005.

Previous data from our lab showed neuromotor developmental delay in TgDyrk1A mice [15]. Thus to get further insight in the effect of *Dyrk1A* overexpression on early postnatal motor development, TgDyrk1A mice were evaluated at four time points important for motor development: PD7, PD10, PD14 and PD21. TgDyrk1A (n = 32) mice showed a similar pivoting activity compared to wild types (n = 32) (Figure 1A). However, we observed a significant delay in the appearance of walking behavior (F_(7,56)_ = 10.290, P = 0.000, repeated measures ANOVA) (Figure 1B) supporting previous reports. This delay was more apparent at older ages, when the adult walking patterns are fully established. TgDyrk1A also showed a delay in the acquisition of negative geotaxis. At PD7, 21.4% of wild type presented a complete negative geotaxis, compared to only 16.6% of TgDyrk1A mice. However, this impairment was rapidly recovered, so that at PD10, TgDyrk1A mice performed the task even better than the wild type mice (F_(5,42)_ = 11.016, P = 0.000, repeated measures ANOVA, Figure 1C). TgDyrk1A mice were also slower in acquiring climbing ability (Figure 1D). At PD7, 28% of wild type while no TgDyrk1A was able to climb the screen (F_(5,72)_ = 32.266, P = 0.000, repeated measures ANOVA), albeit again at PD14 both genotypes correctly performed the task. In the wire suspension test (Figure 1E), TgDyrk1A remained less time hanging in the wire and they fell down earlier than wild type mice (F_(7,56)_ = 6.875, P = 0.000, repeated measures ANOVA). Finally, no significant alterations in the locomotion pattern between wild type and TgDyrk1A mice were observed in the paw print test at PD21 (data not shown).

### Expression Pattern of Dyrk1A during Early Postnatal Development

We next studied the pattern of expression of Dyrk1A in wild type and TgDyrk1A mice during postnatal development (minimum of 6 mice per genotype, age and experiment). Dyrk1A expression is well characterized during embryonic stages [14], [23] and in the adult mice [12], but there were no data on the postnatal period. While during development Dyrk1A is ubiquitously expressed, in the adult it is specifically expressed in motoneurons of the motor nuclei of the brainstem (i.e. Facial nucleus). Since the motor alterations in DS are established through delays in postnatal motor development, we analyzed the expression of Dyrk1A in the brainstem and spinal cord (SC) at different postnatal days (PD7, PD10, PD14). No differences in the expression pattern of Dyrk1A were detected between genotypes, showing no ectopic expression, but increased Dyrk1A signal intensity in TgDyrk1A mice indicating overexpression of the kinase.

#### Dyrk1A expression at postnatal day 7

**Figure 2:**
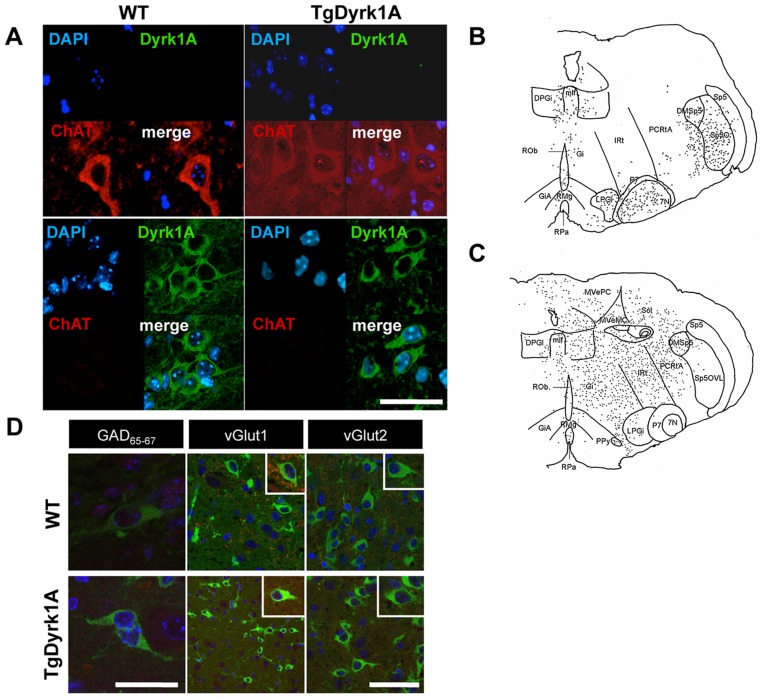
Dyrk1A expression in the brainstem at postnatal day 7. A, D) Collapsed low magnification images of confocal z-stacks of coronal sections (16 µm) of the brainstem from PD7 wild type (left panel) and TgDyrk1A (right panel) mice. A) Double immunofluorescence labeling for Dyrk1A (green) and ChAT (red) and counterstaining with DAPI (blue) showing no positive Dyrk1A cells in MTN of the facial nucleus. Note the typical morphology of ChAT positive MTNs (upper panel). Dyrk1A positive neurons in specific nuclei of the reticular formation located around the facial nucleus (lower panel). B, C) Histological maps showing: B) ChAT positive-Dyrk1A negative neurons mostly localized in the facial motor (VII) and trigeminal motor (V) nuclei and C) Dyrk1A positive neurons in several nuclei of the reticular formation: dorsal nucleus paragigantocellularis (DPGi), gigantocellularis reticular nucleus (Gi), intermediate reticular nucleus (IRT), parvicellular reticular nucleus-alpha part (PCRtA), paragigantocellularis lateral nucleus (LPGi) and in the raphe nuclei (nucleus magnum –RMG- and pale nucleus –RPA). D) Double immunostaining of Dyrk1A (green) and glutamate decarboxylase (GAD_65–67_), vesicular glutamate transporter 1 (vGlut1) and vesicular glutamate transporter 2 (vGlut2). Upper panel show wild type and lower panel TgDyrk1A mice. Inserts represent higher magnification images of selected positive neurons. Scale bar (valid for all panels): 10 µm.

At PD7, immunohistochemistry against choline acetylcholine transferase (ChAT) showed cholinergic neurons mostly in the facial (FN) and trigeminal motor nuclei (Figure 2A,B), although scattered ChAT positive neurons also appeared in other brainstem areas. At this stage, Dyrk1A was not expressed in motoneurons (MTNs) of the FN (Figure 2A, upper panel), but we detected a positive Dyrk1A population in specific nuclei of the reticular formation located around the FN (Figure 2A, lower panel, D): dorsal nucleus paragigantocellular (DPGi), gigantocellular reticular nucleus (Gi), intermediate reticular nucleus (IRT), parvicellular reticular nucleus-alpha part (PCRtA), paragigantocellular lateral nucleus (LPGi) and in the raphe nuclei (nucleus magnum –RMG- and pale nucleus –RPA). Also, Dyrk1A expression was found in the medial longitudinal fascicle (MLF) and nucleus peripyramidal (ppy) (Figure 2C). In the reticular formation, Dyrk1A was not co-localized with calcium binding proteins, serotonin or tyrosine hydroxylase, but we detected co-immunostaining with GABA, GAD_65–67_, vGlut1 and vGlut2 (Figure 2D), suggesting that Dyrk1A is expressed in glutamatergic and GABAergic neurons in both genotypes.

#### Dyrk1A expression at postnatal days 10 and 14

**Figure 3:**
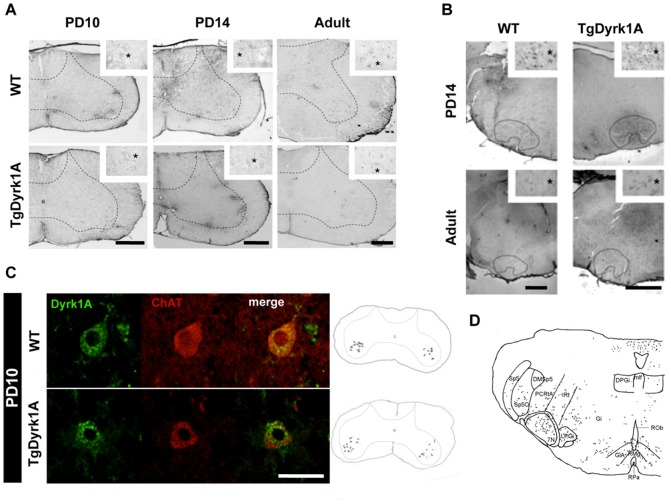
Dyrk1A expression in the spinal cord and facial nucleus at postnatal day 10, postnatal day 14 and adulthood. A) At PD10, Dyrk1A expression started to be detected in MTNs of the spinal cord (left panel), where it remained until adulthood (PD14: middle panel; adult: right panel), in both wild type (upper panel) and TgDyrk1A mice (lower panel). Insert shows MTNs (*) at higher magnification. B) At PD14 (upper panel), Dyrk1A expression disappeared from neurons of the reticular formation and started in the MTNs of the facial nucleus, where it remained until adulthood (lower panel) in wild type (left panel) and TgDyrk1A (right panel). Insert shows Dyrk1A positive MTNs (*) at higher magnification. C) Double immunofluorescence studies at PD10 showed the co-localization of Dyrk1A (green) positive MTNs with ChAT (red) staining in wild type (upper panel) and TgDyrk1A (lower panel) mice that confirmed their cholinergic phenotype. Note the strong Dyrk1A cytoplasmic granular immunostaining in the MTN soma and proximal neurites. Right panel depicts the morphological maps locating Dyrk1A positive neurons in the ventral horn of the spinal cord. D) Mapping of Dyrk1A positive cells showing its location in the MTNs of the FN and other motor nuclei of the brainstem. Abbreviations: DPGi, dorsal nucleus paragigantocellularis; Gi, gigantocellularis reticular nucleus; IRT intermediate reticular nucleus; PCRtA, parvicellular reticular nucleus-alpha part; LPGi, paragigantocellularis lateral nucleus; and raphe nuclei (RMG, nucleus magnum, and RPA, pale nucleus), facial motor (VII) and trigeminal motor (V) nuclei. Scale bar for A and C: 10 µm, Scale bar B: 15 µm.

At PD10, Dyrk1A started to be detected in motoneurons (MTNs) of the spinal cord (SC; cervical, dorsal, lumbar; Figure 3A) and FN (Figure 3B), where it remained until adulthood in both genotypes. Double immunofluorescence studies showed co-localization of Dyrk1A positive MTNs with ChAT, confirming their cholinergic phenotype (Figure 3C, left panel), and their localization in the ventral horn of the SC (Figure 3C, right panel).

From PD10 to PD14, Dyrk1A expression disappeared from neurons of the reticular formation and was detected in the MTNs of the FN (Figure 3B) and other motor nuclei of the brainstem, such as the trigeminal nucleus that were positive for Dyrk1A and ChAT (Figure 3D). However, only 70% of the ChAT positive neurons of the FN at PD14 expressed Dyrk1A in both genotypes, although TgDyrk1A showed increased intensity of immunoreactivity against Dyrk1A.

**Figure 4:**
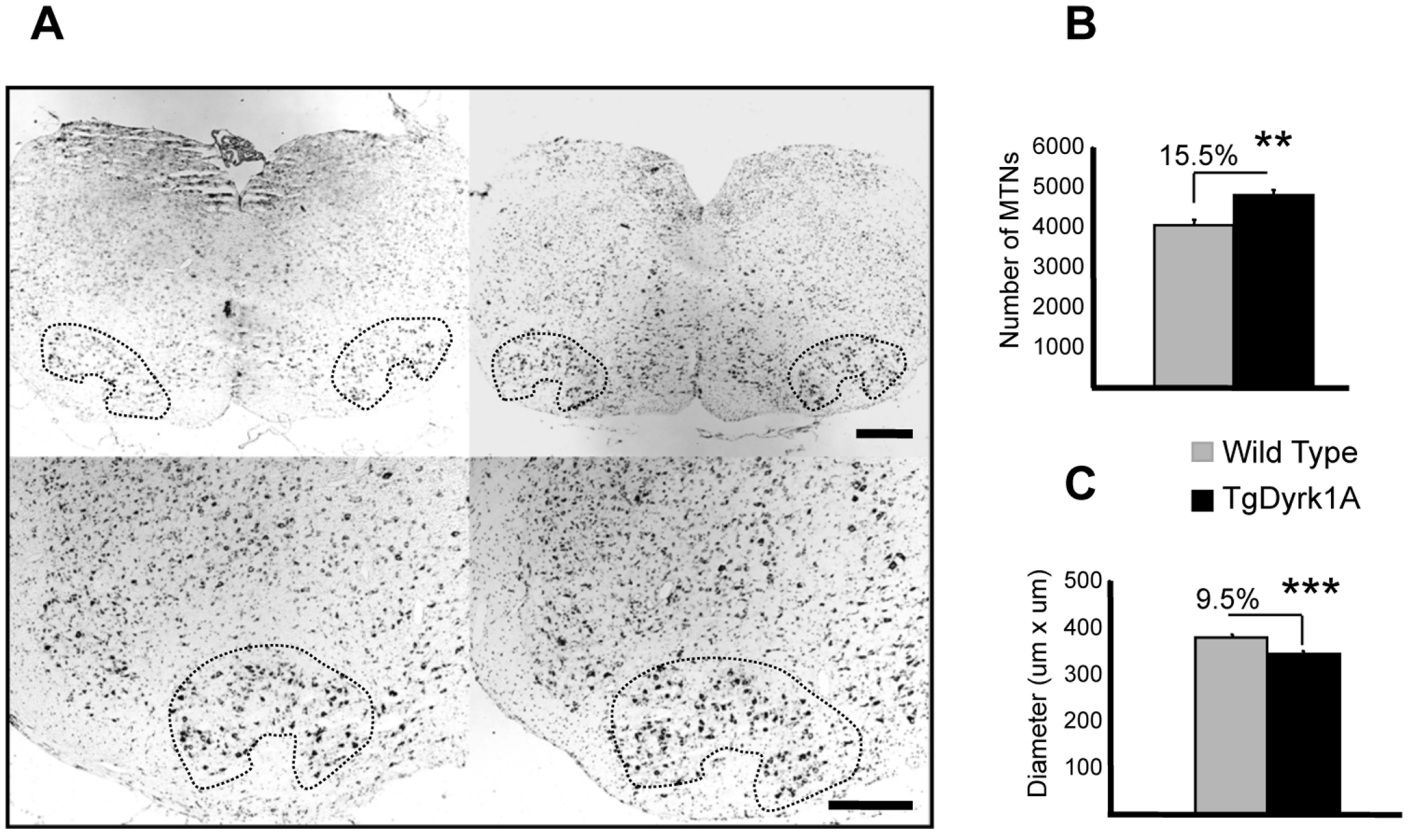
Quantification of the number of motoneurons in the facial nucleus of adult TgDyrk1A. Counting of facial motoneurons was performed on serial Nissl stained paraffin sections (9 µm). A) The number of MTNs in the facial nucleus was quantified in adult wild type (left panel) and TgDyrk1A (right panel) mice. Lower panels show high magnification of the corresponding slice. B) TgDyrk1A mice showed a significant increase of total number of MTNs of the facial nucleus. C) TgDyrk1A MTNs presented a reduction of their diameter. Data in the histograms are represented as means +/− SEM. ** P<0.005, *** P<0.001. Scale bar (for upper panels): 15 µm, (for lower panels): 1 mm.

Motoneurons of the SC and the FN showed a strong cytoplasmic granular Dyrk1A immunostaining in the soma and proximal neurites at PD10 and PD14, completely excluding the nucleus, similar to that previously described during adulthood [12]. Although no important genotype-related differences were observed in Dyrk1A expression pattern during postnatal development, adult TgDyrk1A mice showed a 15.5% of significant increase of total number of MTNs (*t* = −4.145, P = 0.003, Student’s *t* test, Figure 4A,B) in the FN. In addition, MTNs of TgDyrk1A mice had smaller diameters (*t* = 4.461, P = 0.000, Student’s *t* test, Figure 4A,C).

### Dyrk1A Expression at the Neuromuscular Junctions

**Figure 5:**
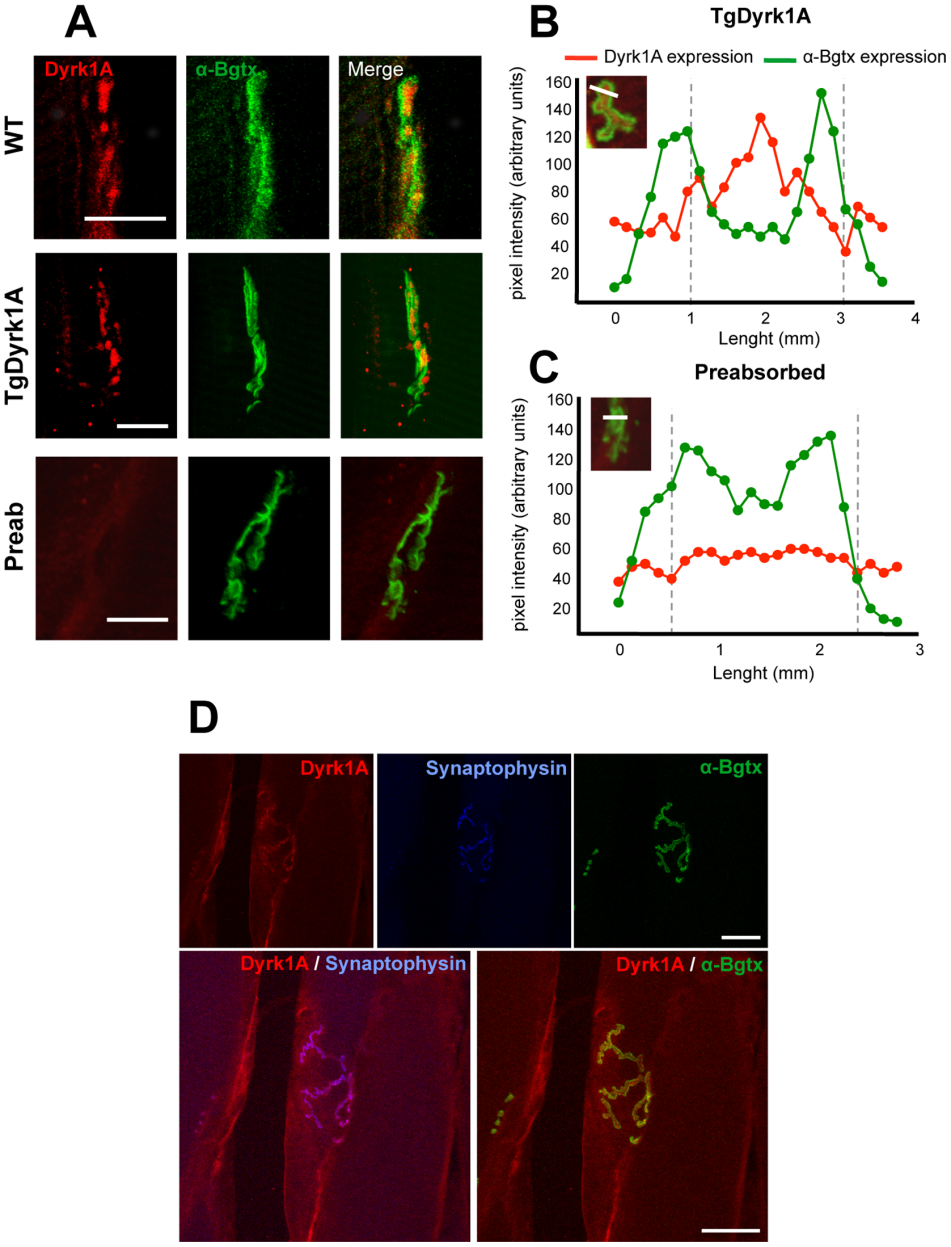
Dyrk1A expression at the neuromuscular junctions. A) Dyrk1A (red) was found in neuromuscular synapses, where it was not co-localized with α-bungarotoxin (αBtx; green) in wild type (upper panel) and TgDyrk1A (middle panel) mice, indicating that Dyrk1A is located in the presynaptic region. Lower panel depicts pre-absorption experiments against mouse anti-Dyrk1A antibody. B-C) Intensity values (arbitrary pixel intensity units) for Dyrk1A (red) and αBtx (green), a marker of acetylcholine receptors, fluorescence were plotted along a line traced over a synaptic fold in individual endplates on (B) TgDyrk1A mice and (C) pre-absorbed antibody. D) Dyrk1A (red, upper left panel) was present at the pre-synaptic site of the neuromuscular junctions, showed by triple fluorescence immunohistochemistry. Synaptophysin (blue, upper central panel) was used as a pre-synaptic marker. α-bungarotoxin (αBtx; green, upper right panel) was used as a post-synpatic marker. Merge images for Dyrk1A and synaptophysin (lower left panel) showed a clear co-localization between both markers. Lower right panel showed only αBtx expression and there was not a co-localization with Dyrk1A. Scale bar: A) 10 µm and D) 25 µm.

Previous work had described the location of Dyrk1A in neurite terminals, and suggested its involvement in neurotransmission at the synaptic site [24], [25]. Thus, we studied Dyrk1A expression at the neuromuscular junctions in adult mice (6 months). Fluorescent α-bungarotoxin (αBtx) conjugate was used to identify postsynaptic ACh receptors, to localize neuromuscular junctions and innervated motor endplates (Figure 5A). Intensity values for Dyrk1A and αBtx fluorescence were plotted for individual endplates on TgDyrk1A mice (Figure 5B). Dyrk1A was not detected with a pre-absorbed anti-Dyrk1A antibody, as a negative control (Figure 5C). To confirm the observation of presynaptic location of Dyrk1A in neuromuscular junctions, a double immunostaining of Dyrk1A and synaptophysin was performed. A clear colocalization was observed, indicating Dyrk1A immunostaining associated with presynaptic terminals but not with the post-synaptic marker (αBtx, Figure 5D).

**Figure 6:**
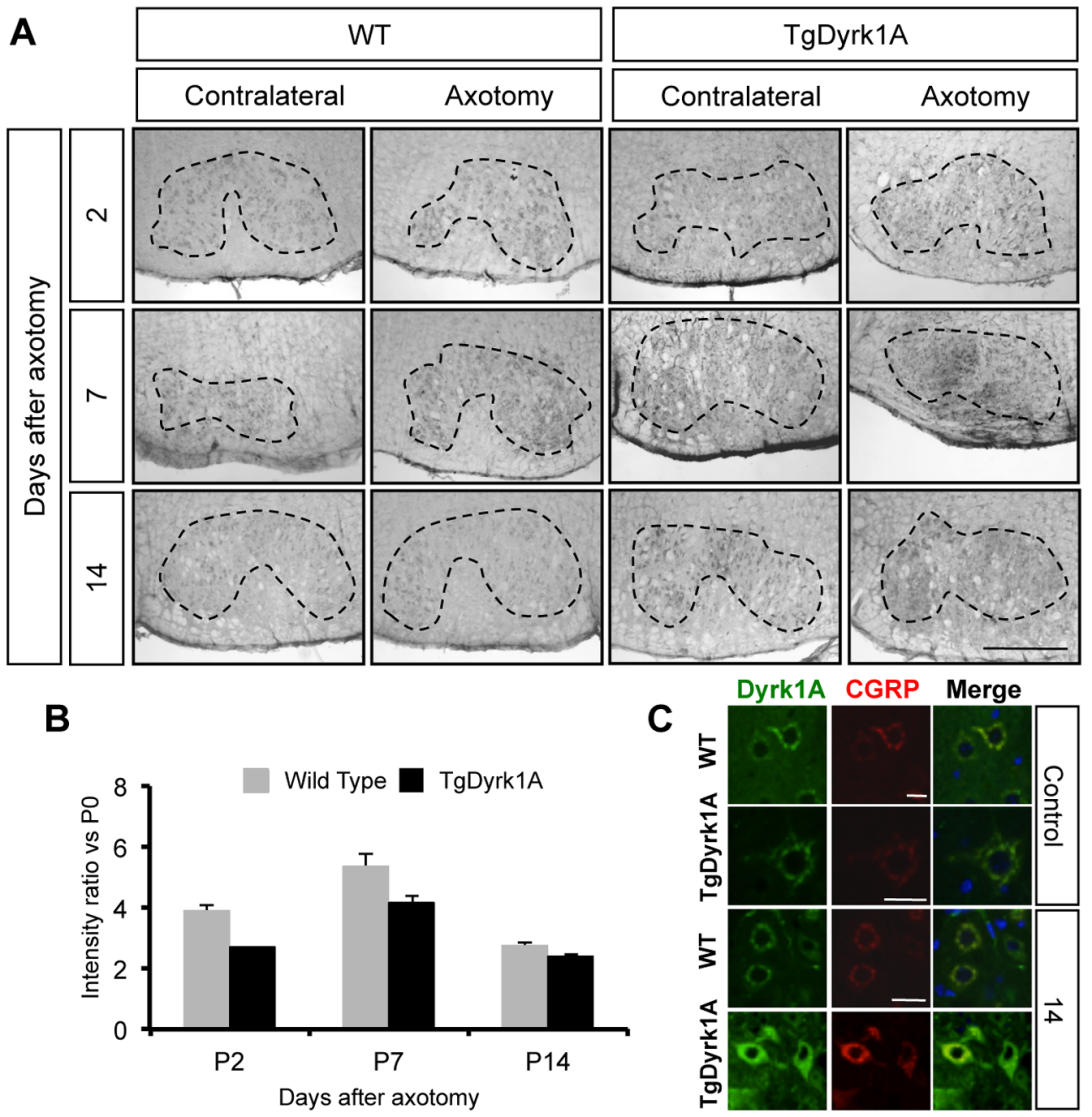
Axonal transport of Dyrk1A in adult mice facial nerve. Axotomies of wild type and TgDyrk1A mice were performed, sacrificing mice at different time points after surgery. A) Expression of Dyrk1A in the facial nucleus was analyzed at different time-points (2, 7, 14 days) post-axotomy in the axotomized and contralateral facial nucleus of wild type (left panel) and TgDyrk1A (right panel) mice. B) The intensity ratio between axotomized and contralateral sides was used to determine Dyrk1A accumulation in the soma as an indicator of the disruption of transport. Expression levels of Dyrk1A in the facial nucleus of wild type and TgDyrk1A mice in non-axotomized conditions (P0) were used for normalizing the ratio at P2, P4 and P14. The expression ratio increased 7 days after surgery as compared to the non-axotomized condition, and was reduced at day 14th post-axotomy in both genotypes. C) To verify Dyrk1A accumulation in the soma of motoneurons after axotomy, we analyzed its co-expression of CGRP. Upper panel shows Dyrk1A (green), CGRP (red) and merge of both in wild type and TgDyrk1A in non-axotomized animals, called basal/control group. 14 days after surgery (lower panel), both genotypes showed an increase of Dyrk1A-CGRP co-expression. Data in the histograms are represented as means +/− SEM. Scale bar for A 15 µm. Scale bar C: upper panel: 55 µm; second upper panel: 60 µm; third upper panel: 50 µm.

In order to analyze the possible axonal transport of Dyrk1A protein, we performed unilateral facial nerve axotomies in adult mice. The animals were then sacrificed at different time points after surgery (days 2, 7, 14) to determine Dyrk1A accumulation in the FN (Figure 6A). A group of non-axotomized wild type and TgDyrk1A animals were used to determine the basal levels of Dyrk1A expression in the FN. In the non-axotomized group, the expression level of Dyrk1A was higher in TgDyrk1A mice as compared with wild types (*t* = −2.937, P = 0.043, Student’s *t* test). For the axotomized groups, the ratio between axotomized and contralateral sides was calculated in each time point after axotomy and ratios were normalized to the basal Dyrk1A expression levels of each genotype.

The ratio of Dyrk1A expression between axotomized and contralateral sides increased 7 days after axotomy and was decreased by day 14 postaxotomy in both genotypes (Figure 6A,B). These results suggested that Dyrk1A is transported along the axons to the neuromuscular junctions since after axotomy Dyrk1A was accumulated in the soma of the FN MTNs. To verify this Dyrk1A accumulation, we analyzed the expression of calcitonin gene related peptide (CGRP), a neuropeptide located in granular vesicles of the Golgi apparatus and smooth endoplasmic reticulum of MTNs [26], at 14 days after surgery. CGRP accumulates in the soma of MTNs 15 hours after axotomy of the FN, reaching a peak at 6 days after surgery, and gradually decreasing to normal levels [27]. In our experiments, Dyrk1A co-localized with CGRP and both genotypes showed a concomitant increase in CGRP expression (Figure 6C).

## Discussion

Down syndrome (DS) individuals present important motor deficits that have been classically ascribed to structural and functional deficits in the cerebellum [28]. Genetic dissection studies in mouse models showed the implication of *Dyrk1A,* a candidate gene for DS abnormalities, in the adult DS motor phenotypes [12], [16], [17], [20], [29], [30], [31], [32]. However, even though DS motor deficits are the consequence of altered motor development of infants and young children, there are very few studies addressing its pathogenetic mechanisms in this extremely important life period. Here we have explored the postnatal expression pattern of *Dyrk1A* in the brainstem and spinal cord and impact of its overexpression during postnatal motor development in transgenic mice (TgDyrk1A). Our results confirmed and extended previous studies [15] indicating a delayed cranio-caudal maturation of the motor system. Dyrk1A showed a time-dependent postnatal expression in specific brainstem nuclei of the reticular formation, and later expression in motoneurons (MTNs) of the facial motor nucleus (FN) and the spinal cord. Importantly, we describe for the first time the presence of Dyrk1A in the presynaptic terminal of the neuromuscular junctions and its axonal transport from the facial nucleus, suggesting a function for Dyrk1A in these structures.

In our experiments, we have confirmed and extended previous observations indicating that Dyrk1A overexpression in TgDyrk1A mice gives rise to neuromotor developmental delay [15]. TgDyrk1A mice showed a delayed development of adult-like walking ability (PD10 and PD14) that was compensated by PD21. The maturation of negative geotaxis required to detecting and to correct body position, was also slightly, though not significantly delayed in TgDyrk1A, suggesting that *Dyrk1A* overexpression could affect propioception and vestibular development. This assumption was reinforced by the significantly slower acquisition of the climbing ability.

In the neonate, immature motor patterns mainly depend on spinal and brainstem control and it is subsequent motor maturation what stems from a growing integration of supraspinal, intraspinal, and sensory control. Previous studies showed ubiquitous spatio-temporal expression of Dyrk1A in the mid/late embryonic (E12–18), the early postnatal (PD0–5) [13], [14] that was restricted to the olfactory bulb, some forebrain regions and MTNs of the brainstem (facial nucleus) and spinal cord in the adult mouse brain [12]. In our experiments, a positive Dyrk1A population was detected in specific nuclei of the reticular formation, such as the dorsal nucleus paragigantocellular, gigantocellular reticular nucleus, intermediate reticular nucleus, parvicellular reticular nucleus-alpha part, paragigantocellular lateral nucleus and raphe nuclei, that disappeared from the reticular formation at PD10. *Dyrk1A* transcripts were previously reported in the basal plate of the rhombencephalon during embryonic period, in several pontine nuclei and in the rhombencephalic reticular formation, as well as in the cochlear and vestibular nuclei of the alar plate [13], [14]. Our study suggests that its expression persists during early postnatal stages, both in glutamatergic and GABAergic neurons as shown by its co-localization with GABA, GAD_65–67_, vGlut1 and vGlut2.

At PD10, a period that is crucial for the development of motor patterns such as walking ability, Dyrk1A expression started in MTNs of the facial nucleus and spinal cord (cervical, dorsal, lumbar), where it persisted until adulthood and was co-localized with ChAT. Dyrk1A showed a strong cytoplasmic granular immunostaining in the soma and proximal neurites at PD10 and PD14, completely excluding the nucleus, similar to the subcellular pattern previously described [12].

Interestingly, the number of FN MTNs was significantly increased in TgDyrk1A mice although they were smaller than in wild type mice. *Dyrk1A* has been suggested to control cell proliferation and exit from the cell cycle at precise locations along the neural tube [23]. In a YAC transgenic mouse line (152F7) overexpressing *DYRK1A*, increased brain weight and neuronal size were detected, along with increase in phosphorylation of the transcription factor FKHR and with high levels of cyclin B1 [33]. Our results suggest that Dyrk1A may also affect proliferation of other neuronal populations. However, MTNs’ size was decreased in TgDyrk1A that could be interpreted as receiving less terminals contacts [34].

Motor dysfunction in DS is accompanied by hyporeflexia and reduced muscular strength and tone [4], [35], [36]. Thus, we also studied if Dyrk1A was expressed in at the neuromuscular junctions. We found Dyrk1A in neuromuscular synapses, but it did not co-localized with α-bungarotoxin (αBtx) a post-synaptic marker of acetylcholine receptors (AChRs), indicating that Dyrk1A is located in presynaptic terminals. These results suggested that Dyrk1A could be transported along the axons to the neuromuscular junctions. To proof this hypothesis, we performed facial nerve axotomies, and analyzed expression of Dyrk1A in the FN MTNs. Should Dyrk1A undergo axonal transport, its increased accumulation in the soma would be an indicator of the disruption of transport. In our experiments, the ratio of expression of Dyrk1A between axotomized and contralateral sides was increased 7 days after axotomy in both genotypes, and thereafter reduced at post-axotomy day 14. The expression levels of CGRP, a neuropeptide that accumulates in the soma of motoneurons after axotomy of the facial nerve [27], were also increased after surgery in both wild type and transgenic mice. Interestingly, we observed a co-localization of Dyrk1A with CGRP in granular cytoplasmic vesicles of the MTNs, where it coexists with ChAT, and in the Golgi apparatus and smooth endoplasmic reticulum [26].

In conclusion, we here demonstrate time-specific postnatal Dyrk1A expression in the brainstem, spinal cord and in the neuromuscular junctions where it arrives through anterograde axonal transport. *Dyrk1A* overexpression in TgDyrk1A mice leads delays in motor maturation and increased numbers of small-size MTNs. Interestingly, Dyrk1A was found in the brainstem motor nuclei, which integrity is required for the timely development of motor function. Taken together our work indicates that the role of *Dyrk1A* in motor function may be more complex than previously envisaged and suggest that normalization of its dosage could be a good therapeutic strategy in DS.
